# 2020 - A Significant Year for Nursing

**DOI:** 10.1590/1518-8345.0000.3405

**Published:** 2020-12-09

**Authors:** Barbara Stilwell

**Affiliations:** 1 Former Senior Director of Health Workforce Solutions, IntraHealth International, Chapel Hill, NC, USA and Global Campaign Executive Director for Nursing Now in London, UK.


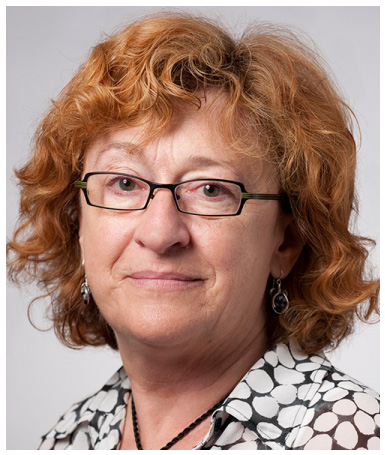


This year was always going to be special for nurses. It marks the bicentenary of Florence Nightingale’s birth and The Nursing Now campaign was planning to commemorate her contribution to nursing with some big events, leading global celebrations with our groups and with our partners, the International Council of Nurses (ICN) and the World Health Organization (WHO). In 2019 The WHO resolved that 2020 would be the Year of the Nurse and the Midwife and in April 2020 published the first ever State of the World’s Nursing Report^(^ 1 ^)^.

Then Covid 19 appeared in all our lives and plans quickly changed. The State of the World’s Nursing - a landmark for nursing - went almost unreported, with little media acknowledgement. Ironically, though, nurses were also in the spotlight all over the world as ‘superheroes’ - front line workers caring for people with Covid 19. Indeed nurses were present at every step of the pathway for diagnosing, treating and recovering from the virus, and for palliative care too. Where nurses were not visible was as decision or policy makers; they were usually not on the platform of Ministry meetings, not speaking to the media as frequently as physicians and not included in scientific advisory committees. Nurses were everywhere - but remained invisible. It was because nurses tend to be invisible, even though they are the largest cadre of the health workforce worldwide, that Nursing Now began its campaign to raise the status and profile of nurses globally.

The story of Nursing Now begins with the Triple Impact report^(^ 2 ^)^ published in 2016. The Triple Impact report about global nursing concluded that a fundamental change was needed in the way that nurses are regarded and treated if they are to be able to play their full role in achieving UHC, and the Nursing Now Campaign built its goals on the strategies recommended in the Triple Impact report. The goals of the Campaign are that there will be:

- increased investment in nursing;
- changes in global policy to enable nurses to practice to the top of their license;
- strengthened nurse leadership and influence;
- better evidence of impact;
- improved ways of sharing effective practice, especially with those who are not nurses.

Underpinning the campaign was the observation by the Triple Impact team *that ‘Nursing can and must take the lead on [raising its profile] but cannot achieve [this] without the support of politicians, policy-makers and non-nursing health leaders’* (p5 Triple Impact). Over many decades, nurse researchers have produced considerable evidence about nursing’s effectiveness in many care settings, and yet it remains challenging to get this evidence to policy makers so that it has an impact on care models.

## Claiming the ‘superhero’

While applause and recognition for nurses has been welcome at such a stressful time in global health history, it will not bring about the real changes that the Nursing Now campaign is calling for. In the life of this campaign, nurses have mobilized themselves across the world: we now have 694 Nursing Now groups (local, national and regional in 126 countries - in all regions of the world. Nursing Now is a social movement that has relied on social media to bring together nurses around the world in a common cause: to tell a new story of nursing^(^ 3 ^)^.

In our new story, nurses are the leading actors rather than the supporting cast that so often is the role allocated to them. It is acknowledged that they are central to sustainable health systems, and there is ample evidence, used by policy makers, that nurses can meet individual and population needs, are fit for the present, and innovative and adaptable for the future. Health ministers invest in nurses - as the State of the World’s Nursing asks them to do - because they understand the evidence of the cost-effectiveness of nursing.

The new story has nurses integrated into every health policy initiative so that they can share their understanding and experience. Nurses can work to design services that are fit for the future - meeting the greatest needs at an affordable cost. In our new story the recommendations of the State of the World’s Nursing report are taken seriously by policy makers so they invest strategically in educating, employing and retaining their nursing workforce and use the evidence generated by nurse researchers to shape their investments.

This may seem like Utopia - but this is our moment. Never before have so many nurses been united around the world as they are now through Nursing Now networks. We have a chance to find our collective voice as nurses and as women. Why should health care be delivered by women and led by men^(^ 4 ^)^? The SOWN has shown us that 90% of the global nursing workforce is female - and women still struggle to have a voice in all sectors, but especially in the health sector. It is not enough to applaud nurses while they remain invisible, uncounted, undervalued and silenced. Nurses need a salary that reflects their skill and knowledge, conditions that are safe and well equipped, a career ladder that takes them into the highest positions in health care and to be integral to the planning of all health service provision. Nothing less will be enough.

This campaign that I direct is called Nursing Now because NOW is the moment that we need to take action. Now, because we have to deliver universal health coverage - health for everyone everywhere; now, because we have earned better pay and recognition through our skills and determination to develop our profession; now, because after 2020 we need to rebuild our health sector in a way that is cost effective and delivers the highest quality, most resilient and sustainable care for everyone. Without nurses, this simply cannot happen.

Let us join in raising our voices together: Nursing Now!
